# Evaluation of a natural compound extracted from *Dolichandrone atrovirens* as a novel antioxidant agent using *Caenorhabditis elegans*

**DOI:** 10.1371/journal.pone.0257702

**Published:** 2021-09-22

**Authors:** Manoj Limbraj Yellurkar, Vibhavana Singh, Vani Sai Prasanna, Pamelika Das, Satheeshkumar Nanjappan, Ravichandiran Velayutham, Somasundaram Arumugam

**Affiliations:** 1 Department of Pharmacology and Toxicology, National Institute of Pharmaceutical Education and Research, (NIPER) Kolkata, Kolkata, West Bengal, India; 2 Department of Pharmacology and Toxicology, National Institute of Pharmaceutical Education and Research, (NIPER) Hajipur, Hajipur, Bihar, India; 3 Department of Natural Products, National Institute of Pharmaceutical Education and Research, (NIPER) Kolkata, Kolkata, West Bengal, India; Institute of medical research and medicinal plant studies, CAMEROON

## Abstract

The compound methyl cinnamoyl catalpol (DAM-1) was isolated from the methanol extract of *Dolichandrone atrovirens*. Studies have already reported the antioxidant activity of *Dolichandrone atrovirens* bark extract, but till date the antioxidant activity of the isolated compound DAM-1, remains unexplored. The endogenous process of reactive oxygen species generation which leads to various degenerative diseases, can be broken down using these exogenous moieties from plant origin, herein this study we sought to evaluate the antioxidant potential of the DAM-1 compound using *Caenorhabditis elegans (C*. *elegans)*, which is the primary model to study the antioxidant activity of compounds. Cytotoxicity assay results showed that DAM-1 treatment in the concentration of 10, 25 and 50 μg/ml has shown 100%, 91%, and 50% survival respectively with overall *p*<0.0001 (treatment v/s control group). 3-(4,5-dimethylthiazol-2-yl)-2,5-diphenyltetrazolium bromide–Formazan (MTT) assay results showed that treatment had better survival rates than the control group at different time intervals i.e. 48 h, and 72 h with *p*<0.01. *Mechanosensation (behavioral study) as well as in vivo study results showed that* at 0 h, 10 μg/ml of DAM-1 treatment showed a better anti-oxidative activity than the control group, 25 and 50 μg/ml of DAM-1 treated groups with *p*<0.001 but at 2.5 h incubation with 10, 25, 50 μg/ml of DAM-1 showed an increased anti-oxidative activity than the control group with *p*<0.001. Thermoresistance assay confirmed that the treatment group had more survival than control group with *p*<0.001. Absorption study of DAM-1 in *C*. *elegans* has shown that the absorption of the drug increases up to 180 mins with a slight decrease after 360 mins and then constant absorption up to 1440 mins. This study paves the way towards the initiative to explore the pharmacological role of DAM-1 in various oxidative stress mediated diseases at molecular levels and the absorption study points out its potential role which could be utilized in the metabolomics and proteomics analysis of this compound in other studies.

## Introduction

The last two decades has seen an increased interest in understanding the dual role of reactive oxygen species (ROS) [1], which can be either harmful or beneficial to living systems [2]. Its beneficial effects involve physiological roles in cellular responses to anoxia, for example in defense against several cellular signaling systems. Another contrasting role of ROS is that, at low concentrations, there is induction of mitogenic response while, at high concentrations, ROS can be important mediators of damage to cell structures, including lipids and membranes, proteins, and nucleic acids, a condition termed as oxidative stress [3]. Oxidative stress also has been implicated in the pathology of many diseases such as inflammation, cancer, diabetes, neurodegenerative disorders, cardiovascular diseases and aging. ROS such as superoxide anions, hydroxyl radical, and nitric oxide, inactivate enzymes damaging intracellular components causing injury through covalent binding and lipid peroxidation. ROS production may happen through the induction of polymorphonuclear cells, regular metabolism processes etc. Smoking of cigarettes, radiation and various pollutions also provoke the body to produce ROS, ROS inactivation can be achieved by activating the endogenous antioxidant enzymes like glutathione peroxidase, superoxide dismutase and catalases and non-enzymatic antioxidants like Se, Nn, Mn and Cu. The hazards occurring due to this oxidative stress can also be blocked with the help of antioxidant exogenous products. Therefore the endogenous anti-oxidant process can be facilitated using antioxidants obtained from herb origin [4]. In recent days people are inclined more towards the use of antioxidants from herbal origin. Plants contain various active constituents which show diversity in their chemical, physical properties as also their pharmacological actions. Flavanoids, phenols, tannins are the active phytochemicals or secondary metabolites of plants. Other naturally produced constituents are carotenoids, alkaloids, cinnamic acids etc. with antioxidant effects. These constituents exhibit their antioxidant acivity via scavenging free rdicals, donating hydrogen or electrons or via metal ion chelation. Additionally the plants are too reported for their antibacterial, antidiabetic and anti-inflammatory effects [5,6].

**Fig 1:**
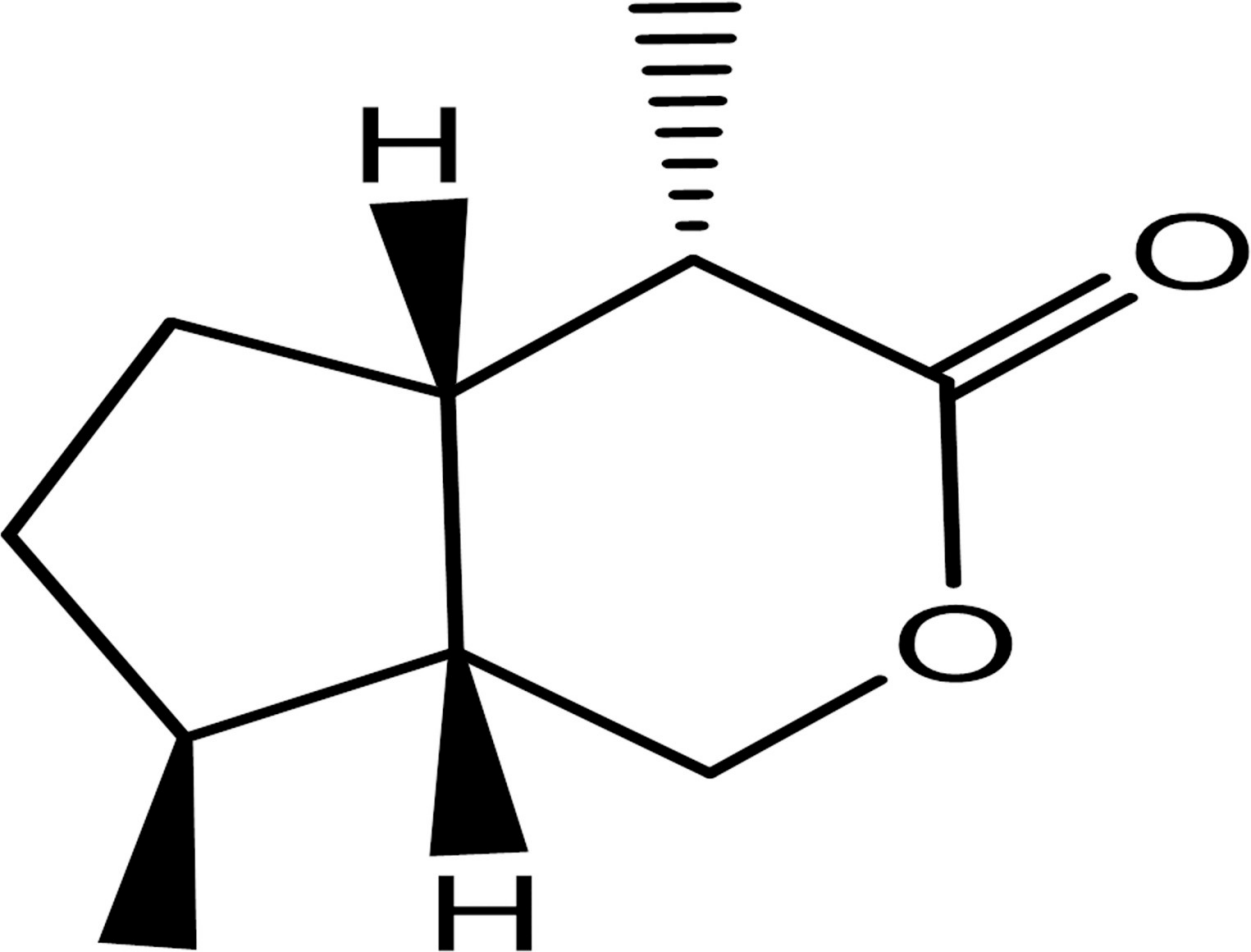
Molecular structures. Structure of iridoid skeleton.

*Dolichandrone atrovirens* (*Bignoniaceae*) is a deciduous tree distributed throughout India and it has long been used by tribes and native medical practitioners to treat various chronic conditions including inflammatory diseases, diabetes, and neurological diseases [7]. It is important to note that only a few phytochemicals may be therapeutically effective among the total constituents present in whole medicinal plant. On the other hand, yield of phytochemicals depends on parts of a plant (i.e. barks, leaves, flowers, roots, fruits or seeds) and their extraction methods [8–13]. DAM-1, methyl cinnamoyl catalpol, is obtained from methanolic extract of *Dolichandrone atrovirens* leaves having iridoid skeleton (type of monoterpenoids) (Fig 1). Iridoids are plants secondary metabolites,which have been reported for their anti-inflammatory, anticancer, neuroprotective, hepatoprotective activity, hypoglycemic activity and hypolipidemic activity [14]. The previously reported data about these class of moieties and novelty of this compound particularly from this plant species and literature review too revealed that no phytochemical and pharmacological studies have been carried out for this compound yet. The various pharmacological study reports of this plants bark and leaf extract in various diseases motivated us to evaluate the antioxidant potential of DAM-1 compound using *C*.*elegans* and various *in vitro and in vivo* assays. The widely used model organism from the nematode category for the antioxidant study is *C*.*elegans*. The reason for using this model is that it is easy to grow using *Escherichia coli (E*.*coli)* as a source of food, its size is very small, rapid reproduction time and short life cycle. The transparent nature of worm and easiest way of genome mutation in this worm has increased its applicability in tracking enzymes and cellular pathways which are involved in aging and oxidative stress using *in vivo* fluorscent markers such as Green fluorscent protein (GFP) [15]. In this research work we studied the antioxidant potential of this compound, which might be helpful to find a novel way in the treatment of many degenerative diseases, developed due to ROS generation mechanism in upcoming days.

## Materials and methods

### *C*. *elegans* and *E*.*coli* strain

Strains used in this study was Bristol N2 (wild-type) *C*.*elegans* worm and the OP50 strain of *E*.*coli* as food source of *C*.*elegans*, which were obtained from the Caenorhabditis Genetics Centre (CGC), University of Minnesota (Minneapolis–St. Paul, MN 55455, United States of America (USA).

### *C*. *elegans* culture

Worms were maintained at 20°C on Nematode Growth Medium (NGM) agar plates prepared as described previously [16], carrying a lawn of *E*.*coli* OP50 as a food source in biological oxygen demand (BOD) incubator. Synchronization of L1 stage worm cultures was achieved by isolating egg from gravid hermaphrodites by treating with the freshly prepared mixture of 4% sodium hypochlorite (Sisco Research Laboratories (SRL), Mumbai, India) and 5 M potassium hydroxide (SRL, Mumbai, India) (1:1 v/v), further vortexed 10 mins for lysis, followed by centrifugation at 1200 rotation per minute (rpm) for 1 min. Supernatant was removed and pellets were mixed with distilled water. Centrifugation step was repeated again at 1200 rpm for 1 min. Supernatant was removed finally and eggs were kept for growing on NGM plates at 20°C in BOD incubator. When these worms reach the L1 stage they were washed several times with M9 buffer before being used for the specified studies [17].

### Vehicle optimization

DAM-1 is soluble in Dimethyl sulphoxide (DMSO) (Sigma Aldrich, USA.), ethanol (Merck millipore, Mumbai, India) or chloroform, (SRL, Mumbai, India.) but the non-volatile vehicle DMSO is preferred over volatile vehicle since the study has a duration period of one week. Vehicle toxicity was studied by taking different concentrations of DMSO i.e 1, 0.9, 0.7, 0.5% v/v in water. Total 10 numbers of of synchronized N2 worms (L1 stage) were added in each well of 24 well microtiter plate along with 100 μl *E*.*coli* OP50 as food source, 400 μl of M9 buffer and 10μl of each solvent percentage (1, 0.9, 0.7, 0.5% v/v of DMSO), which is counted as a treated group while the control group was without solvent treatment. Experiment was done in triplicates. The viability or survivability of nematodes in the vehicles were observed in order to determine a particular non-toxic solvent percentage for future assays [18].

### Sample preparation

1mg of DAM-1 was dissolved in 1 ml of DMSO (0.5% v/v in disilled water) as a stock. Further, the stock was diluted with 0.5% v/v DMSO to make it in the range of 10, 25, 50 μg/ml concentrations of DAM-1 with the help of a sonicator.

### Cytotoxicity assay

For cytotoxicity assay 10 synchronized L1 stage (larvae1) worms were incubated at 20°C in BOD incubator with 400 μl of M9 buffer, 100 μl of *E*. *coli* OP50 culture and 10 ul of different concentrations of DAM-1 i.e 10, 25, 50 μg/ml, for 72 h in each well of 24 well microtiter plate as treated group while the control group was treated with 10 μl of 0.5% v/v DMSO. The survival of worms was determined by touch-provoked movement. Worms were scored as dead when they failed to respond to repeated touching with a platinum wire pick. The test was performed in triplicates [19,20].

### MTT assay

Synchronized L1 stage worms in suspension (500 worms in 50 μl, after validation of volume for no.of worms per μl) were added in triplicate in a 96-well microplate. 10 μl of DAM-1 from 10, 25, 50 μg/ml concentrations was added to this suspension and worms were cultured for 48 and 72 h in BOD incubator at 20°C. Fifty microliters of MTT reagent (10 mg/ml; Sigma Aldrich, USA.) dissolved in filter-sterilized phosphate-buffered saline was added. The plates were incubated at 20°C for 3 h in a BOD incubator, then centrifuged at 800xg for 10 mins, and the supernatant was aspirated. Formazan production was determined 1 h after the addition of 100 μl DMSO (Sigma Aldrich, USA) in the supernatant by reading the absorbance in a microplate reader (Biotek, Synergy HT multimode microplate reader, Winooski, USA) at 575 nm [17].

### Oxidative stress resistance assay (behavioral study)

Ten synchronized L1 stage (larvae 1) worms were added in each well of 24 microtiter plate containing 400 μl of M9 buffer and 100 μl of *E*.*coli* OP50 as a food source and were pretreated with 10 μl from 10, 25 and 50 *μ*g/ml of DAM-1 concentrations and counted as treated group, while another group treated with 10 μl of 0.5% v/v DMSO served as the solvent control group for 72 h, before being exposed to hydrogen peroxide (SRL, Mumbai, India) at 6mM concentration, generating intracellular oxidative stress. Scoring was done based on the viability of nematodes at initial stage and after 2.5 h incubation using stop-frame video analysis software (MAGVISION). Each worm’s behavior is scored by tracing its body movement using MAGVISION, going frame by frame through recording. Experiment was performed in triplicates [19,20].

### Heat shock assays (thermal stress resistant assays)

To assess thermal tolerance, 10 synchronized L-4 stage (adult worms) were transferred in each well of 24 well microtiter plate containing 400 μl of M9 buffer and 100 μl of *E*.*coli* OP50 as a food source and 10 μl from 10, 25, 50 μg/ml DAM-1 concentrations were added serving as the treatment group, while control group was treated with 10 μl of 0.5% v/v DMSO. These groups were incubated upto 72 h in a BOD incubator at 20°C. After incubation with the drug they were placed again for incubation in a BOD incubator at 35°C for 7 h to check the thermal resistance and then scored for viability. The survival of worms was determined by touch-provoked movement. Worms were scored as dead when they failed to respond to repeated touching with a platinum wire pick. Experiment was performed in triplicates [20].

### Absorption study [repeatability and stability of High performance liquid chromatography (HPLC) method]

The Shimadzu prominence LC-20A Ultra fast liquid chromatography (UFLC) (Nishinokyo Kuwabara-cho, Nakagyo-ku, Kyoto 604–8511, Japan) with auto-injector and dual absorbance ultraviolet (UV) detector was used for sample analysis. All samples including standard solutions were filtered through a 0.22-micron polyvinylidene difluoride (PVDF) syringe filter (Merck Millipore, Mumbai, India). Then, 10 μl of each sample was injected into the system and separated in a reverse-phase Agilent C18 column using a mobile phase consisting of 27% of acetonitrile (Rankem, Mumbai, India.) and 73% of methanol (Rankem, Mumbai, India.) with the flow rate of 1 ml/min and the temperature 30°C, detected at 254 nm wavelength. The standard concentration curve of DAM-1 was made first. A series of standard solutions of DAM-1 were made freshly before analysis by diluting the stock (1 mg/ml) in 0.5% DMSO to 10, 20, 30, 40, 50, 60 ug/ml. The calibration curve was plotted with the standard concentrations as the x-axis and the detected peak area signals as the y-axis. The parameters of the slope, intercept and correlation coefficient was carried out by linear regression. Repeatability and stability of method was checked by injecting six replicates of standard with volume 10 μl of 42.4 μg/ml concentration. The retention time (RT) of the drug was observed using HPLC technique [18].

### Absorption study of DAM-1 in *C*.*elegans*

For absorption study, the food source of worms *E*.*coli* OP50 was first grown overnight in a 25 ml conical flask containing 20 ml Luria-Bertani (LB) medium at 37°C in a BOD incubator. On the next day this overnight grown *E*.*coli* OP50 culture was heat-killed at 70°C for 5 mins on water bath [21]. This was used as food source for the entire study. The worms were grown separately in 5 ml tubes at 20°C in BOD incubator as each group in duplicates for each time points starting from 10, 30, 60, 180, 360 and 1440 mins. First group, which contains L-1 stage 500 worms/50 μl (as mentioned above in MTT assay) + 4 ml LB medium + 500 μl of dead *E*.*coli* OP50 as a food source, while second group was containing L-1 stage 500 worms/50 μl + 4 ml LB medium + 500 μl of dead *E*.*coli* as a food source + 100 μl of DAM-1 (42.4 μg/ml). Each sample was harvested using cold M9 buffer at 10, 30, 60, 180, 360, 1080 and 1440 mins intervals and collected in centrifuge tubes. The control group without treatment with the compound was also harvested. The tubes were kept on ice for 10 mins and then spun for 2 mins at 1,150×g to precipitate *C*.*elegans*. The pellets were rinsed three times with cold M9 buffer, air-dried and weighed. The pellets were resuspended using 1 ml of 0.5% v/v DMSO and sonicated 50 times (200 V, operation 5 seconds and stop time 2 seconds). Then, the solution was centrifuged under 12,000×g for 3 mins. The supernatant of the solution containing DAM-1 was transferred to 1.5 ml HPLC vials for injection into the system. The run batch file was made in lab solutions software of Shimadzu (Nishinokyo Kuwabara-cho, Nakagyo-ku, Kyoto 604–8511, Japan) installed in computer sytem connected to UFLC. Sequence of sample loading in batch file was as follows, solvent blank, DAM-1 + *E*.*coli* (duplicates), DAM-1 as standard in 5 replicates and samples in duplicates harvested at each time interval with an injection volume of 10 μl for each samples.

### Statistical analyses

Statistical analysis was performed using GraphPad PRISM software (Graph Pad Software, La Jolla, CA, USA). Data were expressed as mean ± standard deviation (SD). The statistical significance in behavioral and biochemical effects was determined by one-way analysis of variance, followed by a Bonferroni test (post hoc). The results of survival values after stress and toxicity conditions in *C*. *elegans* were analyzed using the *p* test. Differences between the data were considered significant at *p*<0.05.

## Results

### Vehicle optimization

**Fig 2:**
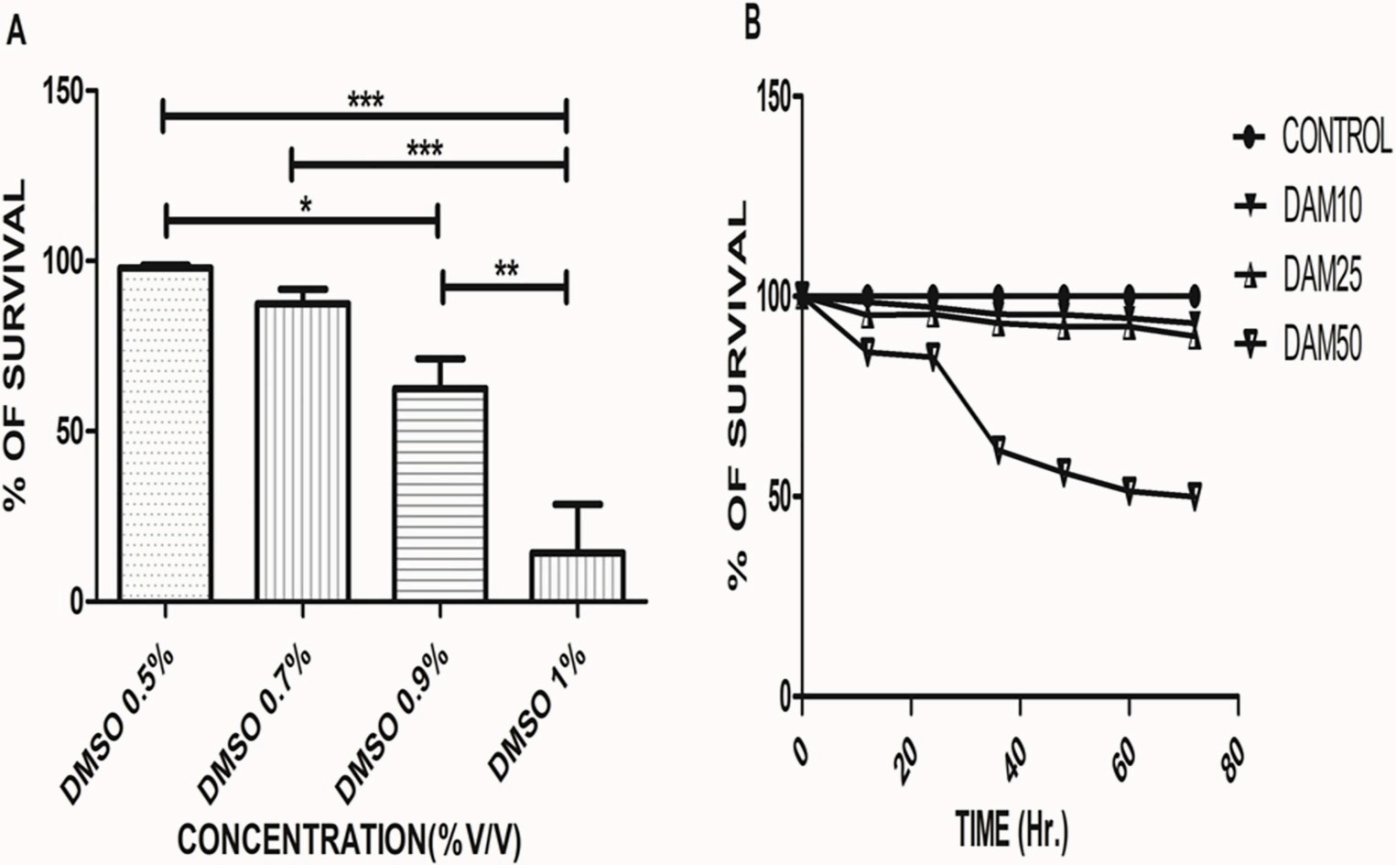
DMSO solvent toxicity assay at different concentrations and cytotoxicity assay of DAM-1. (**A**) Each bar represents % of survival of respected group against treatment in 0.5%, 0.7%, 0.9% and 1% v/v DMSO with mean % ± SD, with *p<0.05 between DMSO 0.5% v/v and DMSO 0.9% v/v, **p<0.01 between DMSO 0.5% v/v and DMSO 0.9% v/v, ***p<0.001 between DMSO 0.5% v/v and DMSO 0.7% v/v. (**B**) The cytotoxicity assay results of DAM-1 in comparison with control in concentrations of 10, 25 and 50 μg/ml with **p<0.001 for DAM 25 vs. DAM 50, ***p<0.0001 for control vs DAM 50 and DAM 10 vs DAM 50.

The results showed that 0.5% v/v DMSO having utmost survival upto 94% at 72 h, while 0.7, 0.9% v/v DMSO has shown survival 70%, 33.66% respectively but 1% v/v DMSO prove to be having survival 0% only. All concentrations were significantly different from each other where *p*<0.05 for 0.5% v/v DMSO vs 0.9% v/v DMSO, *p*< 0.001 for 0.9% v/v DMSO vs 1% v/v DMSO and *p*<0.0001 for 0.5%, 0.7% v/v DMSO vs 1% v/v DMSO (Fig 2A). Thus results from this study suggesting that 0.5% v/v DMSO in water is the optimal non toxic solvent for various study in *C*.*elegans* than 0.7%, 0.9%, 1% v/v DMSO in water.

### Cytotoxicity assay

The results showed that treatment with DAM-1 has better survival rates in a concentration-dependent manner than that of control (0.5% v/v DMSO). DAM-1 treatment in the concentration of 10, 25 and 50 μg/ml has shown 100%, 91% and 50% survival respectively with an overall significance (*p*<0.0001), which indicates that treatment with above mentioned concentration of DAM-1 upto 72h doesn’t have any negative effects on the survival of worms (Fig 2B).

### MTT assay

**Fig 3:**
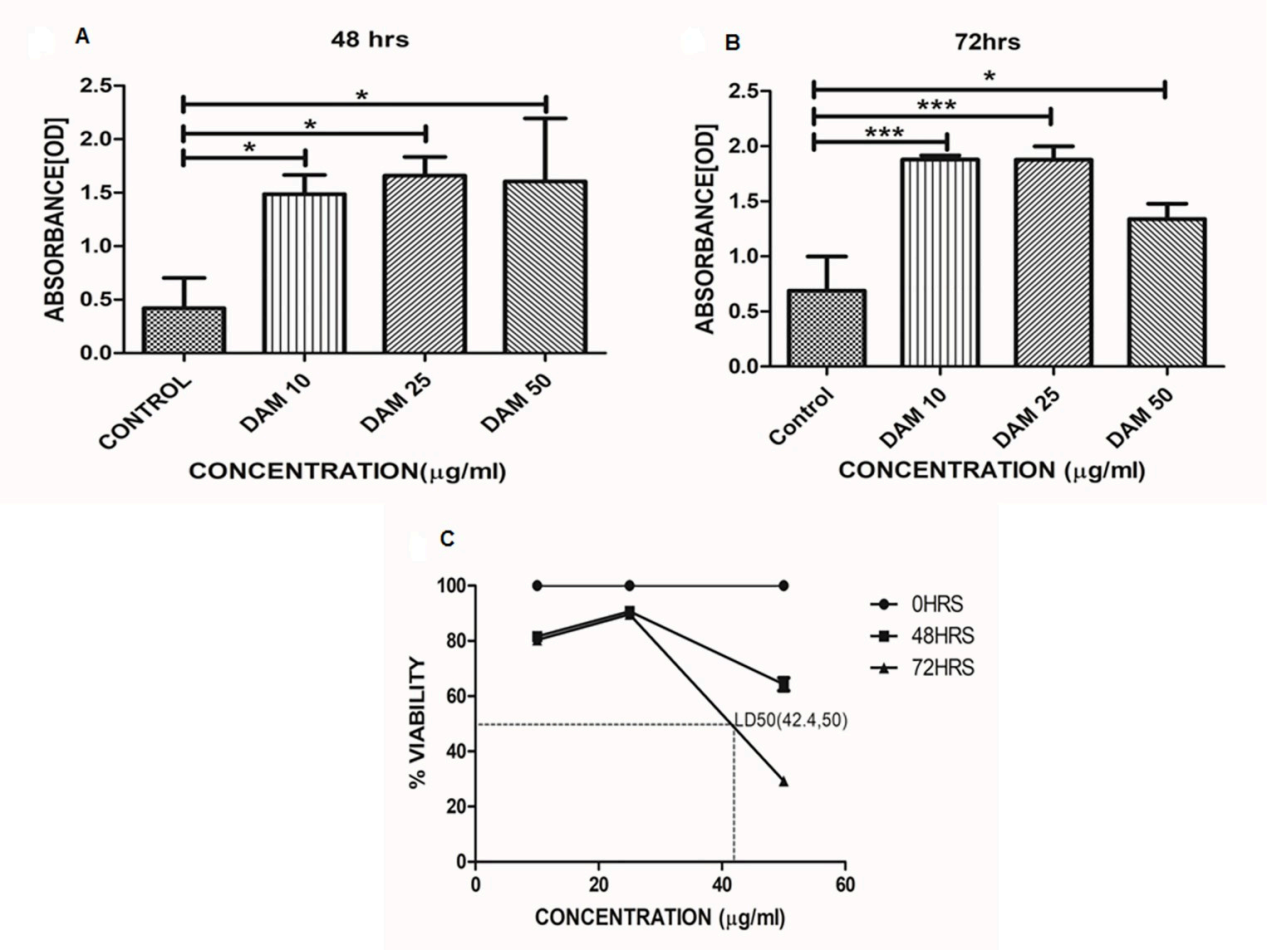
Percentage survival at different time intervals compared to control and LD 50 determination and absorbance [OD] at 48 h, 72 h using MTT assay. (**A**) Percentage survival at different time intervals compared to control against the treatment with DAM-1 has shown LD50 around 42.4 μg/ml. in concentrations 10, 25 and 50 μg/ml. (**B**) Bar represents absorbance in MTT at 48 h with *p<0.01 for control vs DAM 10, 25 and 50 μg/ml (**C**) Bar represents absorbance in MTT at 72 h with *p<0.05 for control vs DAM 50,***p<0.0001 for control vs DAM 25 and 50.

The results have shown that the treatment has more absorbance indicating more production of formazan crystals by live worms from MTT, prompting more survival of worms when compared to that of control group at different time intervals i.e. 48 h (*p*<0.05), 72 h (*p*<0.0001 control vs DAM 10 μg/ml, 25 μg/ml; *p*<0.05 control vs DAM 50 μg/ml) (Fig 3A and 3B). From the results, the (lethal dose 50) LD50 of DAM-1 was calculated as 42.4 μg/ml (Fig 3C).

### Oxidative stress assay

**Fig 4:**
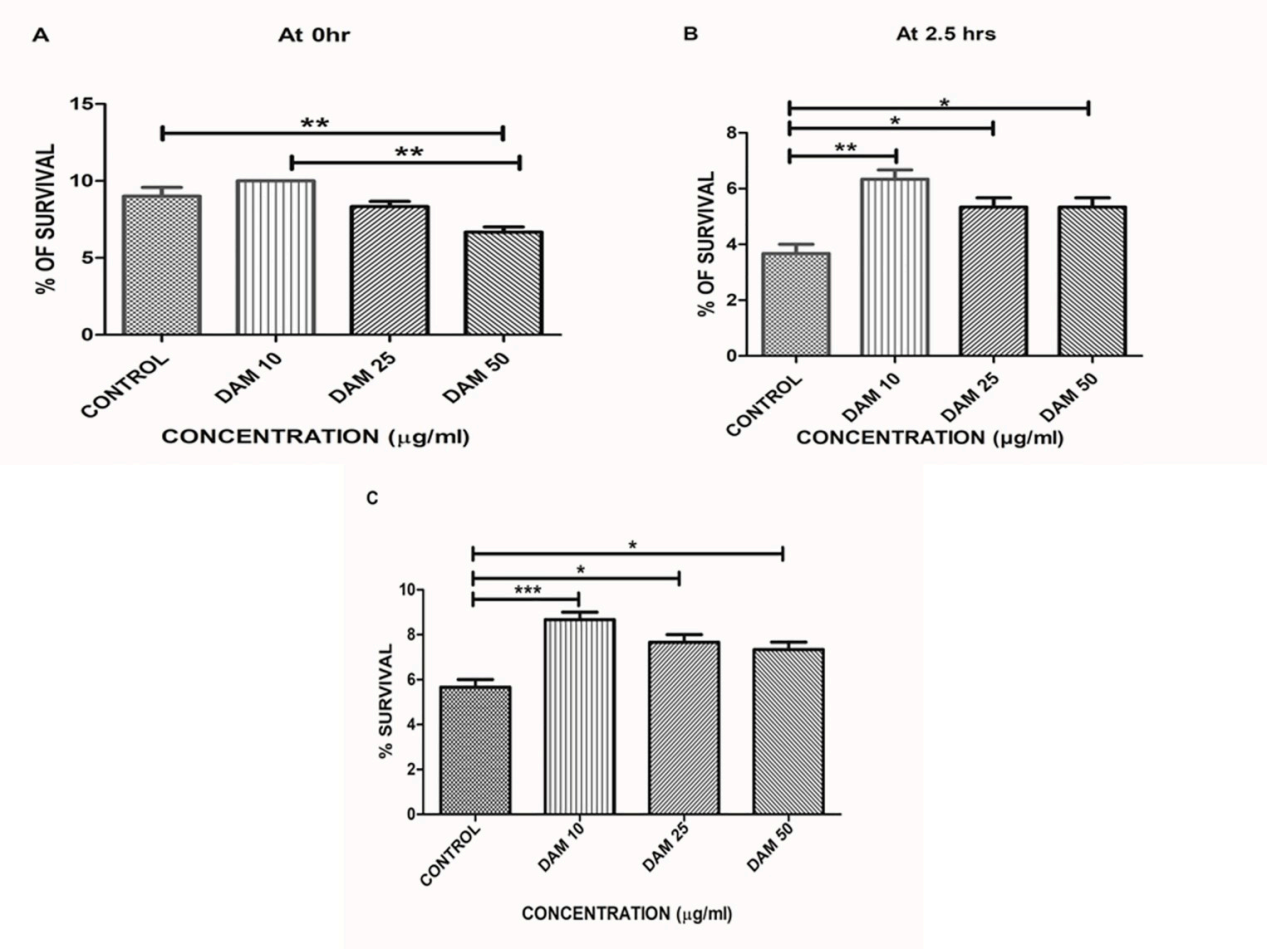
Percentage survival at 0 h, 2.5 h in oxidative stress assay and percentage survival in thermal resistance assay. (**A**) Each bar represent % of survival against treatment with DAM-1 and solvent control in concentrations 10, 25, 50 μg/ml after oxidative stress induction, at 0 h with **p<0.01 for control vs DAM 50 and DAM 10 vs DAM 50. (**B**) Each bar represent % of survival against treatment with DAM-1 and solvent control in concentrations 10, 25, 50 μg/ml after oxidative stress induction, at 2.5 h with *p<0.05 for control vs DAM 25 and 50, **p<0.001 for control vs DAM 10.(**C**) Each bar represent % survival of animals after 7 h in thermal resistance assay with *p<0.05 for control vs DAM 25 and 50 and ***p<0.001 for control vs DAM 10.

*The results showed that 10 μg/ml DAM-1 pretreatment increased the survival rate of worms exposed to hydrogen peroxide induced oxidative stress*, *demonstrating that DAM-1 increases the resistance* to oxidative stress in *C*. *elegans*. It was noted that 25 and 50 *μ*g DAM-1 pretreatments did not enhance the oxidative stress resistance of the worms. 10 *μg/m*l DAM-1 pretreatment increased the survival rate of worms exposed to oxidative stress induced by hydrogen peroxide, with *p*<0.001 control vs DAM 10 μg/ml, 50 μg/ml at initial stage and after 2.5 h time interval, *p*<0.001 control vs DAM 10 μg/ml and *p*<0.05 control vs DAM 25 μg/ml, 50 μg/ml. (Fig 4A and 4B).

### Behavioral parameters of oxidative stress assay

In the behavioral study, we observed that DAM-1 treated worms showed better response (single/successive taps and touching by platinum wire) than the control, after 2.5 h incubation, confirming the anti-oxidative activity (S1A and S1B Table).

### Heat shock assays (thermal stress resistant assays)

The results exhibited that the treatment group have more survival rates than that of the control group, with *p*<0.0001 control vs DAM 10 μg/ml, *p*<0.05 for control vs DAM 25 μg/ml, 50 μg/ml. These findings from results indicate that, the survival of treatment groups have increased due to an increase in thermal resistance of worms than control groups (Fig 4C).

### Repeatability and stability of the HPLC method

**Fig 5:**
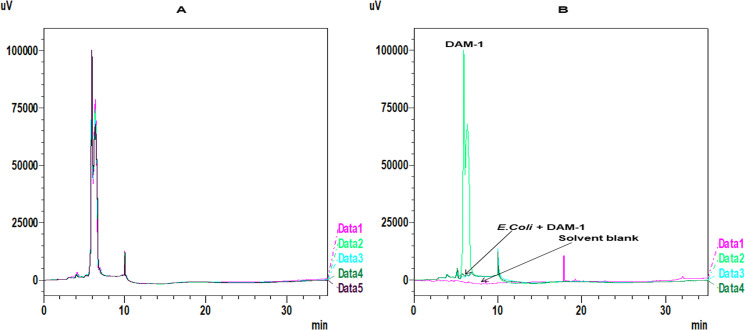
HPLC replicates of DAM-1 as standard and comparison view of DAM-1, *E*.*coli* + DAM-1 and solvent blank. (**A**) HPLC overlay of DAM-1 as standard replicates. Data 1 to 5 as standard replicates 1 to 5 respectively.(**B**) HPLC overlay of DAM-1 (Data 2), *E*.*coli*+DAM-1 (Data 3 and 4), solvent blank (Data 1).

RT of the standard (DAM-1) was 6.15 minutes. The regression equation of the standard curve for DAM-1 was y = 0.09658x+38.546. The linear curve was constructed after repeating the experiment in triplicates with the determination co-efficiency of r^2^ = 0.9992 for DAM-1. This calibration curve showed excellent linearity in the concentration range of (10–60 μg/ml) for DAM-1. The relative standard deviation (RSD) of the six repeated injections of DAM-1 absorption efficiency was 1.02% (Table 1, Fig 5A)

**Table 1:** Repeatability and stability of the HPLC method for six replicates of DAM-1.

DAM-1 replicate no.	Wt. of worm (mg)	% Peak area
1	30	77.567
2	30	77.968
3	30	78.567
4	30	79.569
5	30	77.567
6	30	77.57
	Standard deviation	0.804405536
	% Relative standard deviation	1.029511701

### Absorption of DAM-1 in *C*.*elegans*

**Fig 6:**
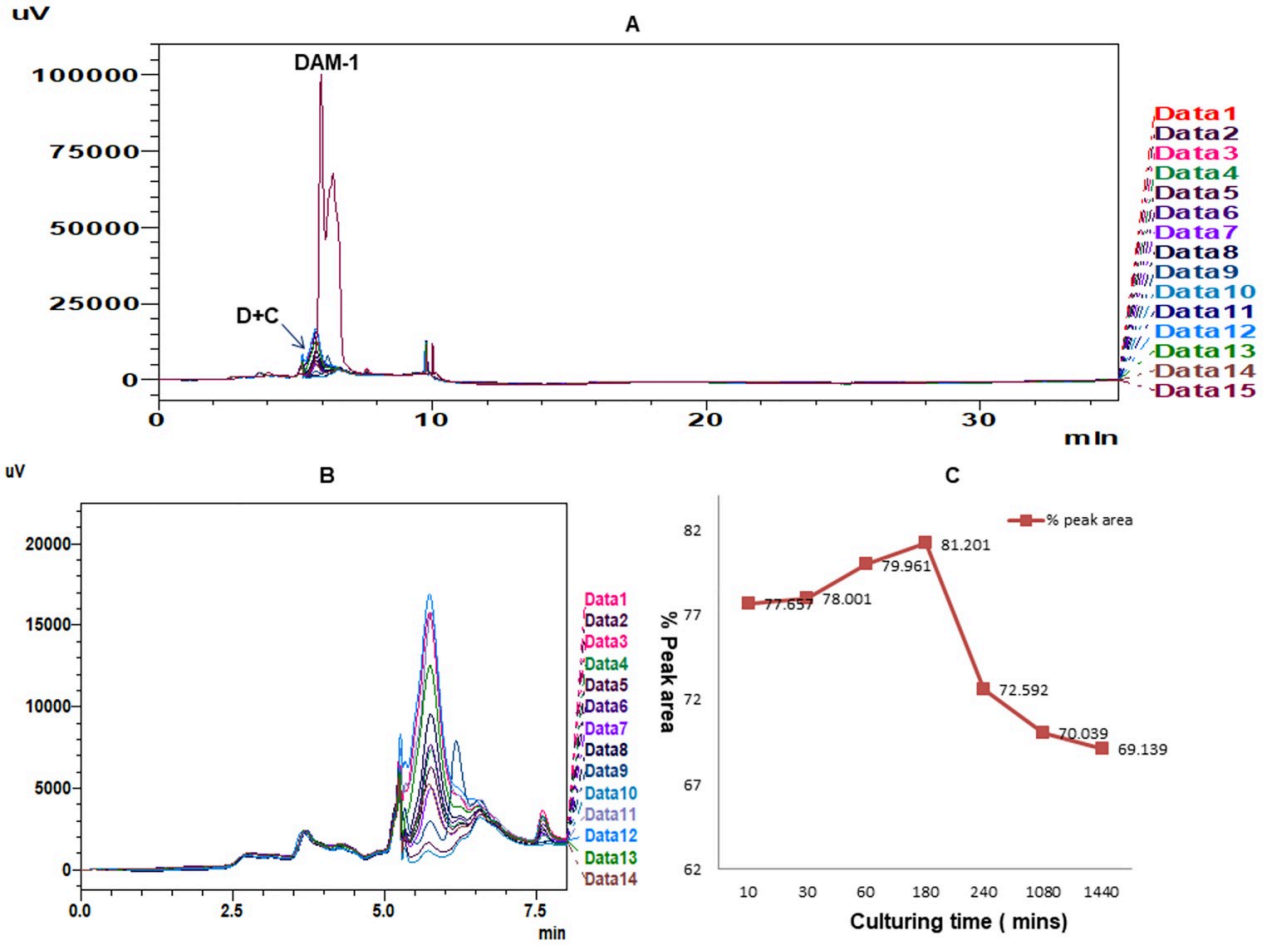
HPLC overlay view of absorption efficiencies of DAM-1 in *C*.*elegans* along with DAM-1 standard and without standard for different culturing time 10, 30, 60, 180, 360, 1080 and 1440 mins at RT 6.15 and graph of % peak area vs culturing time. **[A]** HPLC overlay view of absorption efficiencies of DAM-1 in *C*.*elegans* (D+C, Data 1–14) along with DAM-1 standard (DAM-1, Data 15), **[B]** Zoomed view of DAM-1 in *C*.*elegans* (Data 1–14) without standard peak comparison for different culturing time 10, 30, 60, 180, 360, 1080 and 1440 mins at RT 6.15.

The results from study has shown that the absorption of the drug increases up to 180 mins, but slight decrease in absorbance is noticed after 360 mins with constant absorption up to 1440 mins at RT 6.15 (Table 2, Fig 6A–6C).We have confirmed the presence or absence of DAM-1 in *E*.*coli* sample and solvent blank by comparing these two peaks with standard DAM-1 peak, which showed the absence of DAM-1 in *E*.*coli* sample and solvent blank (Fig 5B).

**Table 2:** Absorption efficiency of DAM-1 in *C*.*elegans* at different culturing time 10 mins, 30 mins, 60 mins, 180 mins, 360 mins, 1080 mins, 1440 mins.

S. No.	Culturing time at different time intervals (mins)	Injected volume (μl)	Worm weight (mg)	% Peak area
1.	10	10	30	77.657±0.772
2.	30	10	30	78.001±0.058
3.	60	10	30	79.961±0.108
4.	180	10	30	81.201±0.066
5.	360	10	30	72.353±0.239
6.	1080	10	30	70.039±0.085
7.	1440	10	30	69.139±0.020

## Discussion

The alternative system of medicines like Ayurveda, Siddha, Unani and other ancestral folklore medicines which has been significantly contributing to the health care of the population of India and nowadays, these systems are not only complementary but also effective in the treatment of various diseases. Diabetes mellitus (DM), which is characterized by hyperglycaemia, is an endocrine, metabolic & chronic disorder resulting from insulin deficiency that leads to high blood glucose concentration due to the metabolism of proteins, fats, and carbohydrates [22–24]. In recent years, high oxidative stress in diabetic patients serves as a major factor in contributing to its complications, such as hypertension, Alzheimer’s disease, chronic kidney diseases [25–27], as it results in excessive production of several advanced glycation end products (AGEs), reactive sugars such as Methylglyoxals (MGOs) and free radicals overwhelms a cell’s natural antioxidant defense, leading to tissue injury or apoptosis [28–31]. It is now becoming remarkably apparent that the available anti-oxidative drugs do not properly meet the therapeutic demands of a vast majority of patients with diabetic health problems, and that herbal remedies remain the ultimate therapeutic hope for many such patients worldwide. Plants have served as a good source of medicine for the treatment of oxidative stress-related diseases. Several studies have been conducted on herbs under a massive amount of ethnobotanical grounds and likewise, a large number of plants possessing anti-oxidative as well as anti-diabetic activities have been noticed. However, till now very little attention has been paid to develop functionally active anti-diabetic drugs from those plants.

Earlier *in vitro* studies demonstrated that bark and leaf extracts of *Dolichandrone* species show antioxidant as well as anti-diabetic activities, by inhibiting various enzymes such as α- glucosidase, α- amylase & glucose 6-phosphatase. As also, it can alleviate hepatic and renal toxicity. Moreover, it is noted that the molecular structure of DAM-1 is similar to that of iridoids, and it is an unknown, pure and isolated compound from whole extract of *Dolichandrone atrovirens*. Iridoids are compounds found in plants that have diverse beneficial, biochemical, anti-diabetic, anti-microbial, anti-septic, anti-oxidant, anti-inflammatory, anti-spasmodic, anti-diuretic, anti-rheumatism as well as anti-cancer activities. The molecular structure of DAM-1 having an iridoid skeleton suggests that, DAM-1 might possess a biological function similar to that of iridoids. In the present study, a DMSO vehicle optimization assay was performed and the results showed that 0.5% v/v DMSO treatment did not affect the survival of *C*. *elegans* up to 72 h. Whereas, 0.7% v/v and 0.9% v/v DMSO showed reduced survival, but 1% v/v DMSO proved to be extremely toxic comparatively. Therefore, we considered 0.5% v/v DMSO as a vehicle for our further experimental studies as it is having a higher survival rate comparatively (Fig 2A). Further, the results from the cytotoxicity assay showed that treatment with DAM-1, ensured better survival in a concentration-dependent manner than that of control (0.5% v/v DMSO). DAM-1 in concentration (10, 25 and 50 μg/ml) has shown 100%, 91% and 50% survival respectively with overall significance *p*<0.0001 (Fig 2B). Therefore, it was concluded that DAM-1 is not cytotoxic at the above-mentioned concentrations.

Oxidative and thermal resistance assays also showed similar results in which, DAM-1 (10 μg/ml) treatment showed better survival than DAM-1 (25 and 50 μg/ml) (Fig 4A and 4B). Also, thermotolerance was better in DAM-1 treated *C*. *elegans* than that of control (Fig 4C). These results suggest that a low dose of DAM-1 (10 μg/ml) significantly improved oxidative stress and provided thermal resistance, whereas DAM-1 at high doses might be toxic to *C*.*elegans*. It is possible that by acting as a mild stress stimulus, DAM-1 activates an adaptive response leading to an increase in resistance of *C*. *elegans* to subsequent stress and at the same time increasing maintenance, repair, and resistance processes.

In the behavioral study, we observed that the DAM-1 treated worms showed better response (single/successive taps and touching by platinum wire) than the control ones after 2.5 h incubation, confirming the anti-oxidative activity (S1A and S1B Table). In the MTT assay, the survival at different time intervals was compared to control (initial, after 48 h, after 72 h) and the effect of drug treatment on *C*.*elegans* was evaluated. Absorbance [OD] was also calculated at different time intervals. The results depicted that, the treatment is having more survival than that of the control group at different time intervals i.e. 48 h and 72 h. The LD50 value reported here is about 42.4 μg/ml, the concentration at which survival of half of the population is inhibited (Fig 3A–3C).

In the present study, DAM-1 treatment is found to be an effective antioxidant in *C*. *elegans*. To aid in exploring this area more in molecular, qualitative and quantitative way, to develop a method for studying the metabolomics and proteomics of this drug in *C*.*elegans* as the future approach, we performed the DAM-1 absorption assay in *C*. *elegans* using HPLC analysis. DAM-1 was administrated with LB agar liquid and *E*.*coli* OP50 dead method (LB agar + dead *E*. *coli*), and thereafter the concentration of the drug in *C*. *elegans* was determined. In results, it was found that the RT of DAM-1 is 6.15 mins and the absorption of the drug increases up to 180 mins, after that the absorption decreases after 360 mins but there is constant absorption up to 1440 mins at RT 6.15 mins. (Fig 6A–6C). Therefore, it was concluded that *C*. *elegans* absorbs DAM-1 efficiently. To investigate other activities and molecular mechanisms involved in antioxidant activity of DAM-1, DAF-16/Forkhead box protein O (FOXO) and SKN-1/ Nuclear factor erythroid 2-related factor 2 (NRF-2) pathways can be studied against DAM-1 treatment. Gene DAF-16 from *C*.*elegans* is homolog of human protein FOXO, whereas, gene SKN-1 is homologus to human NRF2 transcription factors and both are reported to play a protective role against oxidative stress in *C*.*elegans*. The mechanism through which DAF-16/FOXO pathway show antioxidant effects in *C*.*elegans* is via the activation of superoxide dismutase-3 (SOD-3) gene, which is an oxidative response gene and protect the worms from oxidative stress. SKN-1 transcription factor regulates the gene glutathione S-transferase 4 (GST-4), which is an isoform of GST and has key functions in second phase of detoxification process [32–33]. Hence, literature review along with our reported data indicate that, DAM-1 can be a potential drug therapy with antioxidant activity and can be used effectively to treat diseases involving excess ROS generation.

## Conclusion

In conclusion, our study demonstrated that DAM-1, a novel compound obtained from the methanolic extract of *Dolichandrone atrovirens* leaves, have no cytotoxic properties in the selected concentration range, while different levels of antioxidant activity conferred an increase in thermotolerance and oxidative stress resistance in *C*.*elegans* when treated with DAM-1. The absorption study using HPLC revealed a time-dependent increase in absorption of DAM-1. The HPLC data can be used henceforth, in the metabolomics and proteomics study of DAM-1 using *C*.*elegans* in future. Confirmatory study using higher organisms can aid our findings and can translate the research to some disease-specific model which can pave the way to the development of some effective treatment approach.

## Supporting information

S1 Table Behavioral observations during the assay at a different time intervals after treating with hydrogen peroxide in concentration range of (6mM), test drug (T) in concentration range of (10–50 μg) and vehicle control (C) **A]** Initial or 0 h: (Precipice Response and Fast Motility Common), **B]** Incubation for 2.5 h (response to a single tap, trains of taps and touching by platinum wire).
(DOCX)
