# Supplementary material for: Evaluation of a natural compound extracted from Dolichandrone atrovirens as a novel antioxidant agent using Caenorhabditis elegans
Source: PLoS One. 2021 Sep 22;16(9):e0257702. doi: 10.1371/journal.pone.0257702 (PMC8457486; doi:10.1371/journal.pone.0257702)
Supplement: S1 Table — Behavioral observations during the assay at a different time intervals after treating with hydrogen peroxide in concentration range of (6mM), test drug (T) in concentration range of (10–50 μg) and vehicle control (C) A] Initial or 0 h: (Precipice Response and Fast Motility Common), B] Incubation for 2.5 h (response to a single tap, trains of taps and touching by platinum wire). (DOCX) [file pone.0257702.s001.docx]

**S1 Table. Behavioral observations during the assay at a different time of intervals**

| 1. Initial or 0 h**: (Precipice Response and Fast Motility Common)** | |
| --- | --- |
| **Test/control** | Behavior on Treatment with 6 mM Hydrogen peroxide |
| **T10** | Fast movement |
| **T25** | Few of them were showing fast movement |
| **T50** | Fast movement |
| **C** | Fast movement |
| **B)** Incubation for 2.5 hrs **(response to a single tap, trains of taps and touching by platinum wire)** | |
| **Test/control** | Behavior on Treatment with 6 mM Hydrogen peroxide |
| **T10** | Movement on single tap |
| **T25** | No movement on single tap but curved and moved after successive tapping/touch |
| **T50** | Slight movement |
| **C** | No movement with slight response |

**C** = Control, **T** = Test
